# Impact of Symptomatic COVID-19 on the Oral Health of Pediatric Patients in Tbilisi City

**DOI:** 10.3390/children12060725

**Published:** 2025-05-31

**Authors:** Lia Mania, Ketevan Nanobashvili, Tinatin Manjavidze, Mamuka Benashvili, Nino Bzishvili, Ia Astamadze

**Affiliations:** 1 Doctoral Program of Public Health, School of Health Sciences, University of Georgia, Kostava Str. 77a, Tbilisi 0171, Georgia; k.nanobashvili@ug.edu.ge (K.N.); t.manjavidze@ug.edu.ge (T.M.); 2 Department of Healthcare, Tbilisi Humanitarian University, Monk Gabriel Salos Ave. No. 31, Tbilisi 0144, Georgia; 3 Department of Medical Statistics, NCDC (National Center for Disease Control and Public Health), Isani-Samgori District, Kakheti Highway 99, Tbilisi 0109, Georgia; 4 Business Statistic Department, National Statistics Office of Georgia, Tsotne Dadiani Str. 30, Tbilisi 0180, Georgia; 5 Clinic NeoLab, 2b Varlam Topuria Str., Tbilisi 0186, Georgia

**Keywords:** oral microbiome, oral health indicators, population-based research, COVID-19

## Abstract

Background/Purpose: Coronavirus disease 2019 (COVID-19) has become the cause of a global health crisis during the pandemic. This research aimed to study the impact of symptomatic COVID-19 on children’s oral health indices and salivary microbiome composition during the post-COVID-19 period. Methods: An observational, cross-sectional study was conducted in Tbilisi (Georgia) among children aged 7–12 years. A total of 421 children included in the study had a history of laboratory-confirmed COVID-19 within one year of exposure. No participants met the criteria for comorbid conditions or for PCC. A stratified simple random selection of schools and among selected clusters was used. The selected children were divided into two groups: the exposed group, who were patients with a history of symptomatic COVID-19, and the control group, who were patients with a history of asymptomatic COVID-19. The data were collected from August 2022 to December 2023. Oral screening, microbiological examination of saliva, and administration of questionnaires were also performed. Logistic regression was used to calculate ORs with 95% confidence intervals. The statistical processing of the data was performed with SPSS 23.0. This study was approved by the Biomedical Research Ethical Council of the University of Georgia (UGREC–04–22/9 March 2022). Results: Statistically significant differences in the means of the oral health indicators between the studied groups were detected (exposed: DMFT + deft = 5.9; MGI = 0.92; S-OHI = 1.9; control: DMFT + deft = 3.8; MGI = 0.56; S-OHI = 1.4). According to the logistic regression, symptomatic COVID-19 had a significant effect on the following oral health indicators: DMFT + deft (OR = 1.26; 95% CI = 1.14–1.39), MGI (OR = 2.31; 95% CI = 1.50–3.55), and S-OHI (OR = 3.43; 95% CI = 2.03–5.76). The effect of symptomatic COVID-19 on the frequency of eradication of the studied microbiome was also significant (OR = 2.12; 95% CI = 1.23–3.63). Conclusions: A close association was established between symptomatic COVID-19 and microbiome changes in the oral saliva of children, as well as between oral health indicators and symptomatic COVID-19. Considering the research results, it is assumed that a symptomatic course of COVID-19 may be an additional risk factor associated with poor oral health in the pediatric population in the post-COVID-19 period.

## 1. Introduction

Georgia is a country in the Caucasus region. Tbilisi is the capital city, where the population as of 1 January 2023 is 1,241,700 people. The first confirmed case of COVID-19 in Georgia was recorded on 26 February 2020. According to the latest data, as of 15 July 2022, 1,673,160 cases of the disease had been officially confirmed in Georgia [1]. By 1 July 2022, according to NCDC data, the number of test-confirmed cases in the population aged 0–18 in Georgia was 294,649 children (17.7% of all confirmed cases); of these, 115,137 children with COVID-19 were recorded in Tbilisi in this age group [1].

Specialized testing to detect COVID-19 in Georgia began on 4 February 2020. As of 1 July 2022, the testing rate (PCR + antigen) was 4897 per 1000 inhabitants. Georgia ranks 10th among the 50 countries in the world with the highest testing rate [1].

The most common symptoms of COVID-19 in children are fever, chills, cough, and sore throat [2,3,4,5].

Among the local symptoms of the oral cavity in the pediatric population, the most often observed were taste and smell disorders, oral candidiasis, gingivitis, Cheilitis simplex, Lingua pilacta, and inflammation of the salivary glands [6].

According to statistics described in the literature, at least one oral symptom was observed in two-thirds of COVID-19 patients [7]. There is evidence that a significant proportion of patients with oral symptoms have various oral pathologies three months after hospital discharge, suggesting that these changes may be a consequence of COVID-19 [8,9].

The oral cavity is also interesting because it is known as the gateway for SARS-CoV-2 infection [9]. The oral mucosa and salivary glands are rich in angiotensin-converting protein 2 (ACE-2) and transmembrane protease serine 2 (TMPRSS2) receptors. The virus directly binds to these receptors and can also damage the salivary glands. Among the symptoms are observed inflammation of salivary glands, sialadenitis, xerostomia, and altered taste [10,11]. These events are pathogenically related to changes in the microbiome of the oral cavity [12,13]. The oral microbiome changes throughout life and is associated with local (oral) and general diseases [14].

The pediatric population is characterized by an asymptomatic and relatively mild clinical course of COVID-19 [15,16]. The conducted studies revealed differences in laboratory and clinical data between children with symptomatic and asymptomatic courses of COVID-19 [17].

Despite the diversity of related studies, relatively little scientific information is available about the impact of the virus on oral health in school-aged children. We believe that our study sheds light on the oral health outcomes of school-age children following symptomatic COVID-19 transmission, which are noteworthy and require a multidisciplinary approach to virus management.

This research aimed to study the impact of the symptomatic course of COVID-19 on oral health indicators and the salivary microbiome among children aged 7 to 12 years in the post-COVID-19 period in Tbilisi City (Georgia).

## 2. Methods

### 2.1. Study Population

This population-based oral health study included 7- to 12-year-old, laboratory-confirmed post-COVID-19 children within 1 year of exposure from Tbilisi (Georgia). No participants met the criteria for comorbid conditions or post-COVID-19 conditions (PCC). Data collection began in August 2022 and finished in December 2023. A total of 421 children were examined. The post-COVID-19 population was selected from public and private schools in Tbilisi using a stratified simple random sampling method. By 2022–2023, there were 282 general education schools in Tbilisi attended by 155,366 students aged 7–12 years [18].

The number of patients included in the study was determined using a sample size formula. The sample size was determined based on the principle that the margin error of the total results should not exceed 5%, and in detail, the reliability of the research results should be at least 90%.

The following formula was used to calculate the size of the required sample:

$$n=\frac{p\left(1-p\right)*N*Z_{(1+q)/2}^{2}}{p\left(1-p\right)*Z_{\frac{1+q}{2}}^{2}+N*d^{2}}*deff$$

where

n is the sample size (421);
N is the population size (115, 366);
d is the maximum permissible error (5%);
q is the confidence level (90%);

$Z_{\frac{1+q}{2}}^{2}$ is the (1 + q)/2 quantiles of the standard normal distribution;

deff is the importance of the design effect.

The number of qualifying schools and children (421 research subjects) was proportionally distributed among the 5 main municipalities of Tbilisi [18] Table 1.

The selection of schools for each municipality was carried out using simple stratified random sampling supported by SPSS 23.0 software. Additionally, the selection of school grades was carried out using systematic random sampling. In the selected classes, we conducted simple random sampling until a predetermined number of students were collected in the study school.

Using this principle, 27 schools and 421 students were selected. A total of 4214 children and their parents were interviewed, resulting in 421 (9.9% of the total respondents) laboratory-confirmed children aged 7 to 12 years who agreed to participate in the study. According to the survey results, 547 children were infected with laboratory-confirmed COVID-19, which equated to 12.98% of the respondents. Among them, 421 beneficiaries (76.96%) agreed to participate in the study.

### 2.2. Research Design and Procedure

The study was observational and cross-sectional.

### 2.3. Exposure and Outcome Variables

The selected children were divided into two main groups:

1. Exposed group–Symptomatic group: Children with a history of symptomatic COVID-19. (Presence of even one local or general symptom);
2. Control group–Asymptomatic group: Children with a history of asymptomatic COVID-19. (No symptoms). The data were collected via questionnaires and verified via NCDC databases.

Symptoms during COVID-19 infection were studied via questionnaires. The presence/absence of temperature was studied from the general symptoms. Thirteen symptoms have been studied from the perspective of local oral manifestations: facial asymmetry, pain in the mouth, redness on the gums or oral mucosa, bleeding from the gums, rash or ulcer on the oral mucosa, curdled plaques, itching, burning sensation, change in taste, change in smell, hypersalivation, dryness of the mouth, and excessive plaque on the teeth.

The outcome was a change in the salivary microbiome and oral health indicators:

Decayed, Missing, Filled teeth (DMFT) for permanent dentition;
decayed, extracted, and filled teeth (deft) for primary dentition;
DMFT + deft for mixed dentition;
Modified Gingival Index (MGI);
Simplified Oral Health Index (S-OHI).

Five species of pathogenic and conditionally pathogenic microorganisms with average and excessive growth were studied in microbiological samples of saliva: *Staphylococcus aureus*, *Candida albicans*, *Pseudomonas aeruginosa*, *Streptococcus pneumoniae*, and *Staphylococcus epidermalis.*

### 2.4. Assessment of Oral Health Indicators and Saliva

Oral health indicators were evaluated according to the standards provided by WHO. (Supplementary Table S1).

Evaluation of the microbiological study of oral saliva: Biological material (saliva) was collected from the oral cavity, and its transportation and subsequent microbiological research were carried out in the “Clinic Neo-Lab” laboratory based on EUCAST guidelines [19]. For the identification of microbes in the laboratory, the culture method of bacteriological diagnostics was used, which is often called the “gold standard” [20,21]. When choosing the method, its budget ability was taken into account, which is important for population studies in public health.

### 2.5. Research Stages

Stage I—Selection of students for survey in selected schools and classes and telephone interviews with parents of selected children to obtain informed consent. Informed consent documents and questionnaires were left at the school for signature by the guardians of the selected children.

Stage II—The guardians of the selected children completed questionnaires about their condition in the oral cavity; these questionnaires were used to determine their history of COVID-19 and clinical symptoms during infection. In addition, families’ socioeconomic status, children’s behavior, and knowledge regarding oral health, hygiene, and nutrition were examined. The questionnaire was developed based on the basic methods for examining the oral cavity provided by the World Health Organization [22]. After the caregivers signed the informed consent document and filled out the questionnaires, the third stage of the study—screening—began.

Stage III—Children were observed in medical rooms of schools, and biological material (saliva) was collected from the transport soil. The visual examination of the oral cavity included the determination of the simplified oral hygiene index (OHI-S), the caries intensity (DMFt + deft), and the modified gingival index (MGI). The data were collected on a dental card (form N 4–220). Only a disposable dental mirror was used during the examination of the oral cavity [23]. Dental plaques were stained with the disclosing agent. During the observation process, an intraoral photo of the oral cavity was compiled both before and after the plaque was stained to increase the reliability of the study [24].

Senior students of the university’s dentistry program participated in the research process. The research team consisted of five researchers: one researcher performed oral cavity observation and plaque staining, the second researcher simultaneously recorded the oral indices on the patient’s medical card, and the third researcher collected biological material (saliva) on the transported soil. The fourth and fifth researchers independently took intraoral dental photographs of each beneficiary. The examination of each beneficiary in the medical room lasted 12–15 min.

### 2.6. Statistical Analysis

Descriptive and inferential statistics were used. The mean values of the oral health indicators MDFT + deft, MGI, and S-OHI and the bacterial species cultivated in saliva were assessed by means of the symptomatic and asymptomatic groups, and the difference between the means was determined using a *t* test. *p* values < 0.05 were considered to indicate statistical significance. Cross-tabulations were used to visualize frequency distributions between variables in the symptomatic and asymptomatic groups. The chi-square test was used to determine the presence of a significant difference between the variables in the groups. In the final part, logistic regression was used to determine odds ratios (ORs), including 95% CIs, to examine the impact of the symptomatic course of COVID-19 on the oral microbiome and oral health outcomes. Data processing was carried out using SPSS 23.0.

### 2.7. Research Ethics (Study Approval)

The ethical approval for this study was given by the Biomedical Research Ethical Council of the School of Health Sciences of the University of Georgia (research code UGREC–04–22/9 March 2022). In accordance with the Declaration of Helsinki, informed consent was obtained from the guardians of the participants in the study prior to inclusion. Research data are confidential. The Ministry of Education and Science of Georgia permitted screenings to be conducted in schools. (Doc code MES 9 22 0000871059).

## 3. Results

Of the 421 children included in the study, 50.4% (n = 212) were female, and 49.6% (n = 209) were male. The mean age of the beneficiaries was 9.8 years (SD = 1.6). A total of 9997 teeth (permanent and primary teeth) were examined from 421 children. A total of 301 children had mixed dentition, and 120 had permanent dentition. For the entire population (n = 421), the average intensity of caries (DMFT + deft) was 5.6. The incidence of caries in the post-COVID population (n = 421) was 89%. It is 69% for permanent teeth and 84% for primary teeth. The prevalence of caries in the symptomatic group reached 92%, whereas that in the asymptomatic group reached 77%. In the symptomatic group, the prevalence of caries in permanent teeth was 71%, and for primary teeth, it was 88%. In the asymptomatic group, the prevalence of caries in permanent teeth was 59%, whereas that in primary teeth was 68% (Table 2).

The mean value of the S-OHI is 1.85, and the mean value of the MGI is 0.86 (Table 2).

Overall, 84.3% (n = 355) of the beneficiaries were allocated to the symptomatic (exposed) group, and 15.7% (n = 66) were allocated to the asymptomatic (control) group. The mean variables of oral health in the symptomatic and asymptomatic groups are shown in Table 2. The *t* test proved that the difference between the means of the oral health indicators in the symptomatic and asymptomatic groups was reliable (*p* < 0.05).

During the research, it was possible to identify a total of 19 microbial species: *Streptococcus oralis*, *Streptococcus mutans*, *Streptococcus mitis*, *Streptococcus salivarius*, *Streptococcus pyogenus*, *Streptococcus pneumoniae*, *Streptococcus sanguinis*, *Streptococcus parasanguinis*, *Streptococcus viridans*, *Staphylococcus hemolyticus*, *Staphylococcus homini*, *Staphylococcus aureus*, *Staphylococcus epidermidis*, *Pseudomonas aeruginosa*, *Rothia dentocariosa*, *Escherichia coli*, *Klebsiella pneumoniae*, *Kocuria kristinae*, and *Candida albicans*.

Our study of the saliva microbiome revealed that five types of microorganisms belonging to the pathogenic and conditionally pathogenic microflora were most often cultured: *Staphylococcus aureus*, *Candida albicans*, *Pseudomonas aeruginosa*, *Streptococcus pneumoniae*, and *Staphylococcus epidermidis.* A total of 70.3% (n = 296) of our population had high growth of one or more of these microbes in their saliva. There was a significant difference between the frequencies of pathogenic microbes studied in the symptomatic and asymptomatic groups according to the chi-square test (*p* = 0.006) (Table 2).

Among the symptomatic children (n = 355), 277 had oral symptoms, and 181 had a temperature consistent with their general symptoms. The most frequently mentioned oral symptoms were taste disorders (26.1%, n = 110), smell disorders (25.2%, n = 106), and excess plaque on the teeth (13.3%, n = 56%) Table 3.

To examine the impact of symptomatic COVID-19 infection on the oral microbiome and oral health indicators, odds ratios were calculated using logistic regression. Symptomatic COVID-19 infection had a significant impact on oral hygiene and the proliferation of pathogenic microorganisms (Table 4).

## 4. Discussion

As the present study showed, the prevalence of caries was significantly greater in the group of children with a history of symptomatic COVID-19 infection than in the group of asymptomatic children. For permanent teeth, the symptomatic group was 71%, and the asymptomatic group was 59%. For primary teeth, the symptomatic group was 88%, and the asymptomatic group was 68%.

In the symptomatic group, the mean DMFT + deft was 5.9, which was evaluated by the WHO as high caries intensity, and in the asymptomatic group, it was 3.8, which was classified as moderate. In the symptomatic group, the mean S-OHI was 1.9, which was considered to indicate fair results, while in the asymptomatic group, the mean hygiene index slightly exceeded the norm (1.4). In the symptomatic group, the mean MGI = 0.92; in the asymptomatic group, the mean MGI = 0.56. Thus, in the symptomatic group, the means of all studied oral health indicators worsened; however, it was shown that a history of the symptomatic course of COVID-19 had a greater impact on caries intensity in the post-COVID population.

In the post-COVID-19 study population, the intensity of caries in primary teeth was greater (3.9) than the intensity of caries in permanent teeth (2.8). A similar trend was observed in the symptomatic and asymptomatic groups. Additionally, the prevalence of caries in primary teeth was greater (84%) than that in permanent teeth (74%). This can be explained by the older age of temporary teeth compared to permanent teeth and their anatomical and morphological features. These results are consistent with those of other studies in which the mean caries score for primary teeth in the post-COVID-19 pediatric population was 5.67, whereas it was 2.53 for permanent teeth [25].

The abundance of the microorganisms studied in this research (*Staphylococcus aureus*, *Candida albicans*, *Pseudomonas aeruginosa*, *Streptococcus pneumoniae*, and *Staphylococcus epidermalis*) has been shown to increase abundantly in the saliva of our population; these microorganisms are pathogenic and conditionally pathogenic, but they are not considered specific causes of caries and periodontal diseases [26,27]. However, their abundant growth significantly disrupts the microbial balance of the oral cavity; therefore, with the symptomatic course of COVID-19 infection, weakening of the body’s general immune system may have a significant negative impact on oral health [28]. The presence of opportunistic oral infections caused by *Candida albicans* in symptomatic COVID-19 patients has been described in other studies [29].

In contrast to these studies, our study showed that, in a group of children with a history of symptomatic COVID-19 infection, the frequency of the five studied microorganism cultures increased, and oral hygiene was significantly worse, similar to other indicators, than in children with a history of asymptomatic COVID-19. However, this study does not allow us to explain in the symptomatic group what the initial trigger for the results obtained was the following: the symptomatic course of COVID-19 had a direct impact on changes in the salivary microbiome; conversely, the symptomatic course of infection aggravated oral hygiene, which led to changes in the salivary microbiome. In addition, our study cannot determine whether symptomatic COVID-19 causes impairment of oral health. To answer such questions and determine cause-and-effect relationships, additional in-depth studies are needed, which, in our case and many other studies, is not possible due to the lack of a control group [30]. Based on the logistic regression results, we can only say that a symptomatic course of COVID-19 infection is associated with worsening oral health in our population.

In general, retrospective studies in the literature have shown that pre-pandemic children had significantly lower caries intensity and more frequent treatment than those registered during the pandemic. Lockdown and sedentarism, violation of nutrition and hygiene regime, and delay of planned dental visits had a significant impact on the oral health of children during the pandemic [31,32].

In our study, 65.8% (n = 277) of the laboratory-confirmed patients had oral symptoms. Oral symptoms were almost equally distributed between the sexes. Similar results were obtained in a study in which a meta-analysis of 35 articles was conducted [33].

Among the oral symptoms, the most frequently reported was a decrease/change in taste (dysgeusia) of 26.1% (n = 110), which is considered to be the most recognized oral symptom of COVID-19 [29,33]. Additionally, smell disorders and an increase in plaque on the teeth were most often observed.

The impact of symptomatic COVID-19 on the severity of carious lesions on permanent and primary teeth was similar, and a slightly greater impact was observed for permanent teeth: DMFT (OR = 1.32; 95% CI = 1.15–1.52) and deft (OR = 1.22; 95% CI = 1.07–1.38). The symptomatic group had a 2.1-fold greater chance of having all five studied microbes (OR = 2.1; 95% CI = 1.23–3.67) and a 3.4-fold greater chance of having impaired oral hygiene (OR = 3.4; 95% CI = 2.03–5.76) than did the asymptomatic group.

Additionally, the symptomatic course of COVID-19 affects periodontal health in MGI patients (OR = 2.31; 95% CI = 1.50–3.55). Thus, by comparing the means, ORs, and logistic regression results, we can conclude that the symptomatic course of COVID-19 infection is an additional risk factor that further worsens the microbial balance of oral saliva and, accordingly, the indicators of oral health.

According to the research methodology, the distribution of the beneficiaries included in the research according to the principle of random selection into groups (symptomatic/asymptomatic) markedly differed: symptomatic, 355 (82.32%), and asymptomatic, 66 (15.67%). A similar trend was observed in the literature review, where a meta-analysis of 48 studies was conducted in the pediatric population of all ages; it was observed that 20% of the children had an asymptomatic course, and the rest had a mild to moderate course [34]. The similar distribution in our population can be explained by the fact that in the country, state funding was used to test only those with a symptomatic history, which is a significant obstacle to the detection of asymptomatic, laboratory-confirmed children. Literature analysis also confirms that a lack of testing in asymptomatic children has also been reported in other countries [35,36].

The beneficiaries included in the study had an asymptomatic, mild to moderate course of infection. Children with a history of severe COVID-19 were not included in the selection, which is explained by the fact that severe COVID-19 infection is rare in the pediatric population [16,37]. Based on the results of the present study, it is possible to hypothesize that in children with severe symptoms of COVID-19, a closer association between the symptomatic course of COVID-19 and oral health in the post-COVID-19 period is expected. The generation of such an idea allows us to recommend a similar study of daisies in the pediatric population with chronic diseases.

Importantly, the present study included clinical and laboratory data to confirm the screening results, and it was not based on subjective data from questionnaires. A scientific innovation is intraoral mobile photography of the beneficiaries conducted in parallel during the screening process.

The impossibility of a deeper microbiological study of oral microflora can be considered a limitation of this research. It should be noted that the PCR study of saliva would have given us the basis for a more detailed analysis and conclusions. However, since the research is not purely clinical and public health provides for budgetary studies aimed at the population, the researchers considered it relevant. The absence of a pre-pandemic control group can also be considered a study limitation.

## 5. Conclusions

Close associations were established between the symptomatic course of COVID-19, oral health indicators (DMF/def, MGI, S-OHI), and changes in the salivary microbiome in the post-COVID-19 period. Considering the research results, it is possible to assume that a symptomatic course of COVID-19 may be an additional risk factor associated with poor oral health in the pediatric population in the post-COVID-19 period.

## Figures and Tables

**Table 1:** Quantitative distribution of qualifying schools and students.

Municipality	Number of the Students	Number of the Schools	Average Number of Students per School Who Participated in the Study	Average Number of Students per Class Who Participated in the Study (6 per Class)
Sum	421	27	16	2–3
Municipality of Gldani-Nadzaladevi	104	6	17	2–3
Municipality of Didube-Chughureti	73	5	15	2–3
Municipality of Vake-Saburtalo	81	5	16	2–3
Municipality of Isani-Samgori	92	6	15	2–3
Municipality of Old Tbilisi	71	5	14	2–3

**Table 2:** Comparison of study groups.

Variable	Level	Groups		Total	Significance
		Symptomatic	Asymptomatic		
Sample		355 (82.32%)	66 (15.67%)	421	
Gender	Female	172 (81.1%)	40 (18.9%)	212 (50.4%)	
	Male	183 (87.6%)	26 (12.4%)	209 (49.6%)	
Age (n)	7	28 (82.4%)	6 (17.6%)	34 (8.1%)	
	8	59 (81.9%)	13 (18.1%)	72 (17.1%)	
	9	71 (84.5%)	13 (15.5%)	84 (20.0%)	
	10	59 (80.8%)	14 (19.2%)	73 (17.3%)	
	11	54 (83.1%)	11 (16.9%)	65 (15.4%)	
	12	84 (90.3%)	9 (9.7%)	93 (22.1%)	
Dentition	Permanent dentition	105 (87.5%)	15 (12.5%)	120 (28.5%)	
	Mixed	250 (83.1%)	51 (16.9%)	301 (71.5%)	
Caries prevalence	Permanent teeth	71%	59%	69%	
	Primary teeth	88%	68%	84%	
	Permanent and Primary teeth	92%	77%	89%	
Teeth (n)	Primary	1965 (83.1%)	400 (16.9%)	2365 (23.7%)	
	Permanent	6464 (84.7%)	1168 (15.3%)	7632 (76.3%)	
		8429 (84.3%)	1568 (15.7%)	9997 (100%)	
DMFT (mean)		3.0	1.7	2.8	*p* = 0.0001 (t)
	D	826 (90.1%)	91 (9.9%)	917 (78.2%)	
	M	10 (90.9%)	<5 (9.1%)	11 (0.9%)	
	F	221 (90.6%)	23 (9.4%)	244 (20.8%)	
	D + M + F(T)			1172 (100%)	
deft (mean)		4.2	2.8	3.9	*p* = 0.002 (t)
	D	774 (87.0%)	116 (13.0%)	890 (75.4%)	
	E	63 (86.3%)	10 (13.7%)	73 (6.2%)	
	F	200 (91.7%)	18 (8.3%)	218 (18.5%)	
	d + e + f(t)			1181	
DMFT + deft (mean)		5.9	3.8	5.6	*p* = 0.0001 (t)
S_OHI (mean)		1.93	1.45	1.85	*p* = 0.0001 (t)
	Good (0.0–1.2)	52 (66.7%)	26 (33.3%)	78 (18.5%)	
	Fair (1.3–3.0)	236 (86.1%)	38 (13.9%)	274 (65.1%)	
	Poor (3.1–6.0)	67 (97.1%)	2 (2.9%)	69 (16.4%)	
				421 (100%)	
MGI (mean)		0.92	0.56	0.86	*p* = 0.0001 (t)
	MGI = 0	99 (75.0%)	33 (25.0%)	132 (31.4%)	
	MGI ≥ 1	256 (88.6%)	33 (11.4%)	289 (68.6%)	
				421 (100%)	
Cultivation of pathogens (n) *Staphylococcus aureus*, *Candida albicans*, *Pseudomonas aeruginosa*, *Streptococcus pneumoniae*, *Staphylococcus epiderma.*	Are not growing	96 (76.8%)	29 (23.2%)	125 (29.7%)	*p* = 0.006 (Chi-Square)
	Growing	259 (87.5%)	37 (12.5%)	296 (70.3%)	
				421 (100%)	
Cultivation of pathogens (mean of 5 species)		1.1	0.7	1.0	*p* = 0.004 (t)
Behaviors related to Hygiene (%)	Good	58 (76.3%)	18 (23.7%)	76 (18.1%)	*p* = 0.03 (Chi-Square)
	Poor	297 (86.1%)	48 (13.9%)	345 (81.9%)	
				421 (100%)	
Behaviors related to sweet consumption (%)	Rarely	49 (76.6%)	15 (23.4%)	64 (15.2%)	*p* = 0.05 (Chi-Square)
	Often	306 (85.7%)	51 (14.3%)	357 (84.8%)	
				421 (100%)	

**Table 3:** Description of symptoms during COVID-19 infection.

	Symptoms	N	%
Oral Symptoms	Facial asymmetry	1	0.2
	Pain in the oral cavity	14	3.3
	Sore/red gums and mucosa	15	3.6
	Bleeding gums	18	4.3
	Rash, ulceration	18	4.3
	Curd plaques on the mucous membrane	14	3.3
	Itching	7	1.7
	Burning sensation	10	2.4
	Loss/change of taste	110	26.1
	Loss/change in sense of smell	106	25.2
	Hypersalivation	15	3.6
	Dry mouth	21	5.0
	Plaque on teeth	56	13.3
	Another symptoms	33	7.8
	None of them	100	23.8
	I do not remember	22	5.2
Oral symptoms (total)		277	65.8
Only oral symptoms		174	41.3
Temperature (total)		181	43.0
Only temperature		78	18.5
Oral symptoms and temperature simultaneously		103	24.4

**Table 4:** Odds ratio calculation using logistic regression.

Outcome	Exposure		
	Symptomatic Course of COVID-19		
	OR	95% CI	
		Lower	Upper
Cultivation of pathogenic microorganisms	2.115	1.233	3.627
S-OHI	3.428	2.037	5.768
MGI_Index	2.309	1.502	3.550
DMFT + deft Index	1.257	1.140	1.386
DMFT_index	1.318	1.146	1.516
deft_index	1.219	1.075	1.384

## Data Availability

Data are contained within the article and Supplementary Materials.

## Supplementary Materials

The following supporting information can be downloaded at https://www.mdpi.com/article/10.3390/children12060725/s1, Supplementary Table S1: Evaluation of oral health indicators.

## References

[1] NCDC COVID-19 in Georgia National Center for Disease Control and Public Health 2020–2022 Report 9th Revision. https://test.ncdc.ge/Handlers/GetFile.ashx?ID=c6c26041-e123-4591-b1c6-50103eb5205f.

[2] WHO Coronavirus Disease (COVID-19). 9 August. https://www.who.int/news-room/fact-sheets/detail/coronavirus-disease-(covid-19).

[3] Bialek S., Gierke R., Hughes M., McNamara L.A., Pilishvili T., Skoff T., CDC COVID-19 Response Team (2020). Coronavirus Disease 2019 in Children—United States, February 12–April 2. MMWR Morb. Mortal. Wkly. Rep..

[4] WHO COVID-19 Disease in Children and Adolescents: Scientific Brief, 29 September 2021. Reference Number: WHO/2019-nCoV/Sci_Brief/Children_and_adolescents/2021.1 COVID-19 Disease in Children and Adolescents: Scientific Brief, 29 September 2021. https://www.who.int/.

[5] Mustafa N.M., ASelim L. (2020). Characterization of COVID-19 Pandemic in Pediatric Age Group: A Systematic Review and Meta-Analysis. J. Clin. Virol..

[6] Nasiri K., Tehrani S., Mohammadikhah M., Banakar M., Alaeddini M., Etemad-Moghadam S., Fernandes GV O., Heboyan A., Imannezhad S., Abbasi F. (2023). Oral manifestations of COVID-19 and its management in pediatric patients: A systematic review and practical guideline. Clin. Exp. Dent. Res..

[7] Lin W., Gao F., Wang X., Qin N., Chen X., Tam K.Y., Zhang C., Zhang M., Sha O. (2023). The oral manifestations and related mechanisms of COVID-19 caused by SARS-CoV-2 infection. Front. Cell. Neurosci..

[8] Gherlone E.F., Polizzi E., Tetè G., De Lorenzo R., Magnaghi C., Rovere Querini P., Ciceri F. (2021). Frequent and Persistent Salivary Gland Ectasia and Oral Disease After COVID-19. J. Dent. Res..

[9] Drozdzik A., Drozdzik M. (2022). Oral Pathology in COVID-19 and SARS-CoV-2 Infection—Molecular Aspects. Int. J. Mol. Sci..

[10] Sakaguchi W., Kubota N., Shimizu T., Saruta J., Fuchida S., Kawata A., Yamamoto Y., Sugimoto M., Yakeishi M., Tsukinoki K. (2020). Existence of SARS-CoV-2 Entry Molecules in the Oral Cavity. Int. J. Mol. Sci..

[11] Sinjari B., D’Ardes D., Santilli M., Rexhepi I., D’Addazio G., Di Carlo P., Chiacchiaretta P., Caputi S., Cipollone F. (2020). SARS-CoV-2 and Oral Manifestation: An Observational, Human Study. J. Clin. Med..

[12] Botros N., Iyer P., Ojcius D.M. (2020). Is there an association between oral health and severity of COVID-19 complications?. Biomed. J..

[13] Tsuchiya H. (2021). Oral Symptoms Associated with COVID-19 and Their Pathogenic Mechanisms: A Literature Review. Dent. J..

[14] Nanobashvili K., Mania L., IvaniSvili R. (2021). Oral Microbiome and Health. Acta Sci. Dent. Sci..

[15] Lu X., Zhang L., Du H., Zhang J., Li Y.Y., Qu J., Zhang W., Wang Y., Bao S., Li Y. (2020). SARS-CoV-2 Infection in Children. N. Engl. J. Med..

[16] Zhang J., Dong X., Liu G., Gao Y. (2022). Risk and Protective Factors for COVID-19 Morbidity, Severity, and Mortality. Clin. Rev. Allergy Immunol..

[17] Yoon S., Li H., Lee K., Hong S., Kim D., Im H., Rah W., Kim E., Cha S., Yang J. (2020). Clinical Characteristics of Asymptomatic and Symptomatic Pediatric Coronavirus Disease 2019 (COVID-19): A Systematic Review. Medicina.

[18] Misintry of Education, Science and Youth of Georgia Ministry of Education, Science and Youth of Georgia (mes.gov.ge). Search System for Schools: სკოლების პორტალი—მთავარი. https://emis.ge/.

[19] European Committee on Antibiotic Susceptibility Testing EUCAST: EUCAST EUCAST: Guidance Documents. Eucast: Guidance Documents. https://www.eucast.org/eucastguidancedocuments.

[20] Samaranayake L.P. (2018). Essential Microbiology for Dentistry.

[21] Walley S. (2012). Essential microbiology for dentistry, 4th edition. Br. Dent. J..

[22] World Health Organization (2013). Oral Health Surveys Basic Methods. Oral Health Surveys Basic Methods.

[23] Irish Oral Health Services Guideline Initiative (2012). Oral Health Assessment: Best Practice Guidance for Providing an Oral Health Assessment Programme for School-Aged Children in Ireland. https://www.lenus.ie/handle/10147/296000.

[24] Estai M., Kanagasingam Y., Mehdizadeh M., Vignarajan J., Norman R., Huang B., Spallek H., Irving M., Arora A., Kruger E. (2022). Mobile photographic screening for dental caries in children: Diagnostic performance compared to unaided visual dental examination. J. Public Health Dent..

[25] Alghamdi S.A., Aljohar A., Almulhim B., Alassaf A., Bhardwaj S.S., Thomas J.T., Almalki A., Aljuaid A.O., Mallineni S.K. (2022). Correlation between BMI and Oral Health Status (DMFT, PI, mSBI, and Salivary 1,5-AG) among the Pediatric Population in Saudi Arabia: A Clinico-Biochemical Study. Children.

[26] Du Q., Ren B., He J., Peng X., Guo Q., Zheng L., Li J., Dai H., Chen V., Zhang L. (2021). *Candida albicans* promotes tooth decay by inducing oral microbial dysbiosis. ISME J..

[27] Rivas Caldas R., Le Gall F., Revert K., Rault G., Virmaux M., Gouriou S., Héry-Arnaud G., Barbier G., Boisramé S. (2015). *Pseudomonas aeruginosa* and Periodontal Pathogens in the Oral Cavity and Lungs of Cystic Fibrosis Patients: A Case—Control Study. J. Clin. Microbiol..

[28] Levinson W. (2016). Review of Medical Microbiology and Immunology.

[29] Cuevas-Gonzalez M.V., Espinosa-Cristóbal L.F., Donohue-Cornejo A., Tovar-Carrillo K.L., Saucedo-Acuña R.A., García-Calderón A.G., Guzmán-Gastelum D.A., Cuevas-Gonzalez J.C. (2021). COVID-19 and its manifestations in the oral cavity: A systematic review. Medicine.

[30] Kumar P., Jat K.R. (2023). Post-COVID-19 Sequelae in Children. Indian J. Pediatr..

[31] Angelopoulou M.V., Seremidi K., Papaioannou W., Gizani S. (2023). Impact of the COVID-19 lockdown on the oral health status of pediatric dental patients in Greece. Int. J. Pediatr. Dent..

[32] Wdowiak-Szymanik A., Wdowiak A., Szymanik P., Grocholewicz K. (2022). Pandemic COVID-19 Influence on Adult’s Oral Hygiene, Dietary Habits and Caries Disease—Literature Review. Int. J. Environ. Res. Public Health.

[33] Iranmanesh B., Khalili M., Amiri R., Zartab H., Aflatoonian M. (2021). Oral manifestations of COVID-19 disease: A review article. Dermatol. Ther..

[34] Cui X., Zhao Z., Zhang T., Guo W., Guo W., Zheng J., Zhang J., Dong C., Na R., Zheng L. (2021). A systematic review and meta-analysis of children with coronavirus disease 2019 (COVID-19). J. Med. Virol..

[35] Gao Z., Xu Y., Sun C., Wang X., Guo Y., Qiu S., Ma K. (2021). A systematic review of asymptomatic infections with COVID-19. J. Microbiol. Immunol. Infect..

[36] Nikolopoulou G.B., Maltezou H.C. (2022). COVID-19 in Children: Where do we Stand?. Arch. Med. Res..

[37] Ludvigsson J.F. (2020). Systematic review of COVID-19 in children shows milder cases and a better prognosis than adults. Acta Pediatr..

