# Supplementary material for: Impact of Symptomatic COVID-19 on the Oral Health of Pediatric Patients in Tbilisi City
Source: Children (Basel). 2025 May 31;12(6):725. doi: 10.3390/children12060725 (PMC12191153; doi:10.3390/children12060725)
Supplement: Supplementary file 1 [file children-12-00725-s001.zip › children-3473374-supplementary.pdf]

Supplementary Table S1: Evaluation of oral health indicators:

DMFT/deft	In the mixed dentition, the DMFT+deft indicator was used and in the permanent dentition, the DMFT. DMFT and deft indices were evaluated by summing up the number of carious/decayed (D,d), missing/extracted (M,e) and filled/restored (F,f) teeth of the examined participants. The average intensity of caries was determined by the ratio of the total scores to the number of examined participants. The intensity of caries was evaluated according to the criteria provided by WHO; 0.0–1.1 points are considered very low, 1.2–2.6 low, 2.7–4.4 medium, 4.5–6.5 high, and > 6.6 very high. ("Oral Health Surveys Basic Methods," 2013) Caries prevalence was calculated as percentage.
S-OHI	Soft and hard plaque on the tooth were assessed by the Simplified Oral Hygiene Index (OHI-S) according to Greene and Vermillion (1964), provided by the WHO. Plaque was evaluated on the vestibular or lingual surfaces of 6 teeth [11,16,26,31,36,46] with a 3-point system: 0 points, absence of plaque; 1 point, the cervical part of the tooth or one third is covered with plaque; 2 points, half of the tooth surface is covered with plaque, and 3 points, more than two-third or the entire tooth is covered with plaque. Soft plaque (debris) (Debris Index [DI]) and hard plaque (calculus) (Calculus Index [CI]) were evaluated separately. The soft plaque index (DI) and calculus index (CI) were obtained by summing the scores and dividing by 6. The simplified oral hygiene index (OHI-S) was calculated by

	summing the obtained debris and calculus indices. The calculated indices were evaluated according to the criteria provided by WHO: good hygiene 0–1.2; average hygiene 1.3–3.0; and poor hygiene 3.0–6.0. (Greene & Vermillion, 1964, WHO.)
MGI	Gingival inflammation was evaluated using the modified gingival index and was defined on the following scale: 0 points, normal gingiva and no inflammation; 1 point, very mild inflammation and slight change in colour and texture, which is not expressed in all parts of the marginal and papillary gingiva; 2 points, mild inflammation, which is expressed in all parts of the marginal and papillary gingiva; 3 points, moderate inflammation, swelling or hypertrophy in the marginal or apical part of the gingiva; and 4 points, severe inflammation with swelling and/or marginal hypertrophy of the gingiva or spontaneous bleeding or ulcer. (Tobias and Spanier, 2020)
